# Functional implications of sleeping little in the wild

**DOI:** 10.1093/sleep/zsaf309

**Published:** 2025-10-04

**Authors:** Niels C Rattenborg

**Affiliations:** Avian Sleep Group, Max Planck Institute for Biological Intelligence, Seewiesen, Germany

Sensory systems enable awake animals to interact with their environment to acquire energy, avoid predators, and find mates. Paradoxically, animals ranging from jellyfish to humans [1, 2] periodically go to sleep—a rapidly reversible state of reduced sensory responsiveness. Many hypotheses have been put forward to explain sleep and its sub-states, slow wave sleep (SWS) and rapid eye movement (REM) sleep, present in mammals, birds, and perhaps some ectothermic animals [2–7]. Broadly speaking, functional hypotheses treat decreased responsiveness as either an adaptive protective behavioral strategy, or a dangerous cost of performing processes supporting adaptive waking neurobehavioral performance that cannot be performed as efficiently, or at all, during wakefulness. In this *Perspective*, I evaluate these explanations for sleep in light of the discovery that some birds and mammals sleep unexpectedly little in the wild (Figure 1).

**Figure 1:**
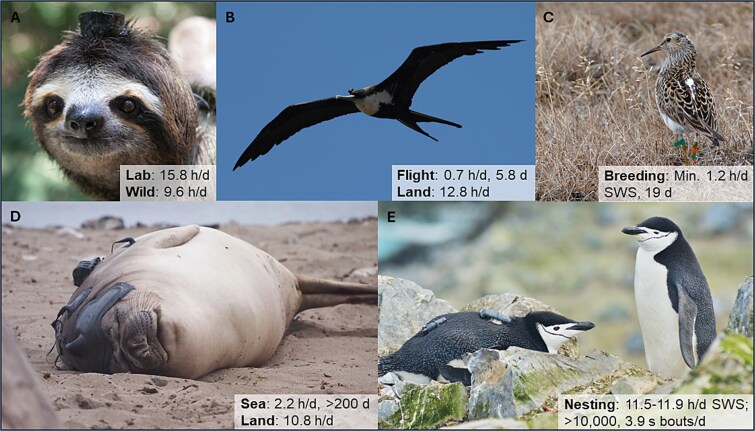
Ecological flexibility in sleep measured with EEG in animals in the wild. (A) Brown-throated sloth recorded in a tropical rainforest in Panama. (B) Great frigatebird recorded over the Pacific Ocean surrounding the Galápagos Islands (Ecuador). (C) Pectoral sandpiper recorded in the Arctic tundra (Alaska, USA). (D) Northern elephant seal recorded along the coast of California (USA). (E) Chinstrap penguins recorded on King George Island, Antarctica. Each animal was equipped with an EEG data logger placed on their head (A, B) or back (C–E). See original papers for details on the EEG loggers and other devices attached to the animals. The insets show the relevant sleep times under various conditions. For C and E, only SWS was measured. Photo credits: (A) Roland kays; (B) Bryson Voirin; (C) Department of Ornithology, MPIBI; (D) Jessica Kendall-Bar; (E) Paul-Antoine Libourel, reproduced with permission from [49].

The immobilization [8] or adaptive inactivity hypothesis [9], suggests that sleep protects animals from predation and conserves energy by preventing them from being active when it would be dangerous or unproductive to be awake. Restorative hypotheses posit that sleep rectifies costly changes occurring in the brain resulting from increased neuronal activity during wakefulness. Sleep is thought to renormalize synaptic strength [10], repair neuronal wear and tear, such as DNA double-strand breaks [11], manage the endoplasmic reticulum stress response to unfolded proteins [12], and remove metabolic waste products, such as β-amyloid, implicated in neurodegenerative diseases [13]. Sleep is also thought to play a role in brain development [14], processing memories [15], regulating emotional reactivity [16], and maintaining the immune system [17]. Many of these hypotheses are not mutually exclusive, and it seems likely that once sleep evolved it took on several functions.

Studies of animals sleeping in the wild provide a new framework for assessing functional hypotheses for sleep. The development of small devices for recording the EEG in freely moving animals has enabled comparative sleep research to move from the artificial lab environment to the wild, where animals face the ecological challenges that shaped the evolution of sleep [18]. In the first EEG-based study of sleep in the wild, three-toed sloths slept 6 h less than in captivity, indicating that the ecological context can have a large impact on sleep duration [19]. By targeting species that live in extreme conditions seemingly incompatible with sleep, researchers also revealed that some animals sleep very little in the wild [20]. Great frigatebirds sleep on the wing, but for less than 1 h per day during 6-day non-stop foraging flights [21]. Sleep in flight is also more unihemispheric, less intense, and more fragmented than sleep on land. Northern elephant seals only sleep 2 h per day while at sea for several months [22]. Some polygamous male pectoral sandpipers sleep 1.2 h per day for 19 days during intense male–male competition for mates [23]. Males that sleep the least sire the most offspring, indicating that sleep loss does not compromise their ability to defend a territory and convince discriminating females to mate. These short sleep strategies pose an important challenge for several functional hypotheses for sleep.

Adaptive sleep loss is difficult to reconcile with restorative hypotheses for sleep. Given that neurobehavioral performance in other animals, such as humans, progressively declines across days of sleep restriction [24], it is a mystery how these animals are able to perform adaptively despite sleeping so little for so long. The immobilization hypothesis seemingly explains this flexibility in sleep duration—animals can simply stay awake when it is ecologically beneficial. However, it does not explain why short-sleeping animals still spend 1–2 h sleeping, despite remaining in the ecological context in which constant wakefulness is seemingly beneficial. More generally, it is unclear why animals sleep, rather than entering torpor, an equally immobile and more unresponsive state with even greater energy savings. The immobilization hypothesis also fails to adequately explain the heterogeneous nature of sleep. Notably, it does not account for animals sleeping deeper or longer after losing sleep. Sleep duration should only reflect the animal’s current ecological demand for wakefulness, not their prior sleep/wake history. The immobilization hypothesis also does not account for the presence of two types of sleep, SWS and REM sleep. The brain warming hypothesis for REM sleep [25, 26] attempts to address this issue by proposing that brain warming during REM sleep [27], likely resulting from the influx of blood in mammals [28] and birds [29], increases brain temperature to waking levels following cooling during SWS, and thereby readies the animal to react rapidly to threats upon awakening [25, 26]. However, it is unclear whether the infrequent awakenings from REM sleep provide sufficient readiness to respond to an attack which can occur at any time. Also, bouts of REM sleep usually terminate before brain temperature reaches waking levels in mice [30] and pigeons [27]. And, nighttime cold exposure reduces sleeping brain temperature by 2°C in European jackdaws, but does not influence the number or duration of REM sleep bouts [31]. The brain warming hypothesis also predicts that REM sleep should increase when animals perceive an increased risk of predation. However, pigeons suppress REM sleep when vulnerable and recover lost REM sleep once safe [32]. Mammals also behave as though REM sleep is dangerous. Notably, horses can engage in NREM while standing, but, due to atonia, need to lie down for REM sleep, a vulnerable position for large herbivores. Indeed, when stressed, horses are reluctant to lie down. The resulting REM sleep deprivation and homeostatic buildup of REM sleep pressure causes REM sleep with atonia to occur while standing, leading to injurious falls [33]. Clearly, in these cases, REM sleep appears to be necessary, but dangerous.

Studies of unihemispheric and asymmetric SWS in birds and marine mammals also suggest that sleep is dangerous and restorative for the brain. Unihemispheric and asymmetric SWS are both associated with sleeping with one eye open, but during unihemispheric SWS the hemisphere contralateral to the open eye is awake, whereas during asymmetric SWS it exhibits EEG slow wave activity intermediate between wakefulness and SWS. Mallard ducks sleeping exposed at the edge of a group keep one eye open and directed away from the group, toward potential threats, whereas ducks flanked by other birds prefer to sleep with both eyes closed and symmetrically with both hemispheres [34]. At sea, northern fur seals sleep unihemispherically, floating on their side with three flippers held out of the water forming a loop resembling a “jug-handle”. The eye facing down into the water stays open, presumably on the lookout for sharks and orca, while the eye facing up is closed [35]. Ironically, indigenous people hunting from canoes with harpoons selectively targeted seals exhibiting this conspicuous “jug-handle” sleep behavior because they were easier to approach than fully awake seals [36]. If sleep provides safety, as the immobilization hypothesis suggests, mallards and fur seals should have evolved a strategy of sleeping with both eyes closed and with both hemispheres when vulnerable. If one concedes that sleep can be dangerous under certain circumstances, but argue that it does not benefit the brain, mallards and fur seals should stay fully awake to maximize their readiness to respond effectively to a predator. Instead, by sleeping asymmetrically or unihemispherically, they appear to mitigate an inherent conflict between the simultaneous need for anti-predator vigilance and sleep functions benefiting the brain. This conflict is also illustrated in European jackdaws experiencing increased sleep pressure, which trade the vigilance afforded by asymmetric SWS for more efficient symmetric SWS [37].

One might argue that the energy saved by sleeping with one hemisphere, rather than any restorative benefits, is sufficient to explain the presence of unihemispheric sleep. However, if the function of unihemispheric sleep were only to conserve energy through lowering the metabolic rate of one hemisphere, then during deprivation of sleep in one hemisphere, the other hemisphere should compensate by sleeping more, as both hemispheres contribute to the animal’s entire energy budget. By contrast, if sleep serves a restorative function for the brain, only the deprived hemisphere should sleep more during recovery. Consistent with the latter, in bottlenose dolphins selectively deprived of deep SWS in only one hemisphere, the deprived hemisphere (1) fell asleep more frequently during deprivation, and (2) usually showed a greater increase in deep SWS time during recovery [38]. Similarly, SWS intensity is homeostatically regulated in a local, use dependent manner in mammalian [39] and avian brains [40]. Consequently, the primary function of unihemispheric SWS is likely restoration for the sleeping hemisphere, rather than energy conservation.

Can we reconcile short sleep strategies with restorative hypotheses for sleep? This depends, in part, on the cause of reduced neurobehavioral performance resulting from sleep loss in humans and other animals. Performance might decline due to increased neuronal damage and reduced restoration. Alternatively, reduced performance might simply result from prophylactic homeostatic processes enforcing sleep before any functional damage can occur [41]. Notably, local sleep (i.e. local slow waves) might account for many of the decrements in waking performance [42–44]. Consequently, preventing local sleep by dampening or forestalling homeostatic processes might be sufficient to account for the preservation of adaptive performance, at least in the short term. This might be possible if other biological drives, such as hunger or reproduction [45], compete with the homeostatic drive for sleep under real-world ecological situations. Finally, as shown for wheel running in laboratory rodents, monotonous behaviors might incur a lower homeostatic demand for sleep [46]. However, in male pectoral sandpipers, short sleep with high performance occurs during a highly dynamic and intense male–male competition for mates that involves establishing a territory, watching for intruding males, available females, and predators, male–male displays and physical fights, aerial and land based courtship displays to choosy females, and foraging for the energy needed to sustain this diverse activity. Arguably, this is the least monotonous and most consequential phase of their life.

Are there long-term costs to a short sleep strategy? Despite sustaining adaptive waking performance, there might be long-term costs of sleeping little. Perhaps short-sleeping animals experience neurodegeneration earlier and die younger than longer-sleeping animals. This live-fast-die-young life history strategy would work if short-sleeping individuals produce as many offspring across their lifespan as longer sleeping individuals. This might explain why short- and long-sleeping male pectoral sandpipers coexist in the population [23]. Short-sleeping animals might also be able to rectify some of the costs of sleeplessness during periods of extended sleep while back on land for frigatebirds [21] and elephant seals [22], and after breeding in sandpipers [23].

Another theoretical option would be to perform sleep functions during wakefulness. The energy allocation hypothesis posits that sleep functions can occur during wakefulness, but under normal conditions it is energetically more efficient to partition them into periods of sleep [47]. Partitioning certain processes during sleep conserves more energy than expected based only on the sleep-related reduction in metabolic rate. However, when ecologically necessary, sleep functions can occur during wakefulness, even without resorting to local sleep, as long as the animal can mobilize sufficient energy to support performing waking and sleeping processes at the same time. Importantly, this would obviate the need for a homeostatic response to increased wakefulness. The appeal of the energy allocation hypothesis is that it reconciles ecological flexibility in sleep duration with restorative hypotheses for sleep. Whether all sleep functions can occur during wakefulness is an open question, however. For example, some types of synaptic scaling and memory processing appear to be mediated by slow waves [10, 15] which also interfere with adaptive waking performance [42–44].

Animals that sleep little in the wild raise several important questions regarding sleep and its functions. Can all of the deficits in waking performance resulting from sleep loss be attributed to the homeostatic intrusion of local sleep? To what extent can other drives, such as motivation, dampen, forestall, or shut off the homeostatic regulation of sleep without causing maladaptive performance? Why are humans vulnerable to falling asleep in life threatening situations, such as driving a motor vehicle, after losing far less sleep than short-sleeping animals? Do short-sleeping animals incur long-term costs, such as shorter lives? Is forgetfulness the price animals pay for wakefulness? Is mobilizing extra energy sufficient for sleep functions to occur during wakefulness? Are there any sleep functions that cannot occur during wakefulness? Is this the essence of sleep?

Finally, in addition to sleeping little, frigatebirds and pectoral sandpipers experience significant sleep fragmentation which can impair waking performance in other animals [48]. The most extreme sleep fragmentation occurs in chinstrap penguins nesting exposed to an egg predator and intraspecific aggression. Despite obtaining large amounts (11.5–12 h per day) of SWS for each hemisphere, they do so via over 10 000, 4-s microsleeps [49]. Are such microsleeps long enough to be restorative? If they are too short, do microsleeps at least minimize neuronal damage by reducing time awake?

Studying unusual animals sleeping in challenging real-world ecological situations has revealed unexpected sleep strategies that challenge commonly held views about sleep. Answering the questions raised by these strategies will likely lead to new perspectives that shape our overall understanding of sleep and the consequences of its loss and fragmentation in animals, including humans.

## Disclosure statement

*Financial disclosure*: None.

*Non-financial disclosure*: None.

## Data Availability

This perspective does not include any data.
